# Group A Streptococcus Infection in Neonatal Population: A Systematic Review of The Literature

**DOI:** 10.3390/jcm12226974

**Published:** 2023-11-08

**Authors:** Rozeta Sokou, Filippos Filippatos, Vasiliki Daniil, Efstathia-Danai Bikouli, Andreas G. Tsantes, Daniele Piovani, Stefanos Bonovas, Zoi Iliodromiti, Theodora Boutsikou, Argirios E. Tsantes, Nicoletta Iacovidou, Aikaterini Konstantinidi

**Affiliations:** 1 Neonatal Intensive Care Unit, “Agios Panteleimon” General Hospital of Nikea, 18454 Piraeus, Greece; danai_mp89@yahoo.gr (E.-D.B.); kmaronia@gmail.com (A.K.); 2 Neonatal Department, National and Kapodistrian University of Athens, Aretaieio Hospital, 11528 Athens, Greece; filipfilippatos@gmail.com (F.F.); vasilikidaniil98@gmail.com (V.D.); ziliodromiti@yahoo.gr (Z.I.); theobtsk@gmail.com (T.B.); niciac58@gmail.com (N.I.); 3 Microbiology Department, “Saint Savvas” Oncology Hospital, 11522 Athens, Greece; andreas.tsantes@yahoo.com; 4 Department of Biomedical Sciences, Humanitas University, Pieve Emanuele, 20090 Milan, Italy; dpiovani@hotmail.com (D.P.); sbonovas@gmail.com (S.B.); 5 IRCCS Humanitas Research Hospital, Rozzano, 20089 Milan, Italy; 6 Laboratory of Haematology and Blood Bank Unit, “Attiko” Hospital, School of Medicine, National and Kapodistrian University of Athens, 12462 Athens, Greece; atsantes@yahoo.com

**Keywords:** group A streptococcus infection, neonatal sepsis, invasive infection, *Streptococcus pyogenes*, perinatal infection

## Abstract

(1) Background: The importance of group A streptococcus (GAS) infection severity has been recognized in children and adults. However, to our knowledge, there have been no systematic reviews or pooled assessments of the incidence and outcome of invasive GAS (iGAS) disease in neonates, a potentially high-risk population. Therefore, we performed a systematic review of available data regarding the risk factors, clinical presentation, and outcome of GAS infection in neonates. (2) Methods: An electronic search of the existing literature was carried out during the period July 2023–September 2023 in the PubMed and Scopus databases, considering studies referring to GAS infection in the neonatal population. (3) Results: Overall, 39 studies met all the inclusion criteria and were included in this review, evaluating data from 194 neonates. Unfortunately, there were a lot of missing data among the retrieved studies. Our systematic review highlighted the presence of differences with regards to clinical presentation, infection sites, and outcome of GAS invasive disease between neonates with early-onset (EOS) or late-onset sepsis (LOS). Common characteristics of EOS included respiratory distress, rapid deterioration, and high mortality rate irrespective of the infection site, while rash, gastrointestinal tract symptoms, and fever appeared to be the most frequent symptoms/clinical signs and manifestations of LOS disease. The management of severe invasive iGAS disease consists mainly of specific antimicrobial treatment as well as supportive care with fluids and electrolyte supplementation, minimizing or counteracting the effects of toxins. Furthermore, a mortality rate of approximately 14% was recorded for iGAS disease in the total of all studies’ neonates. (4) Conclusions: Although iGAS is a rare entity of neonatal infections, the potential severity of the disease and the rapid deterioration requires the development of quick analysis methods for the detection of GAS allowing the prompt diagnosis and administration of the indicated antibiotic treatment. Furthermore, given the exceptional risk for both the pregnant woman and the neonate, it is very important to raise awareness and create easily accessible guidelines that could facilitate the prevention and management of maternal as well as the subsequent neonatal severe iGAS disease.

## 1. Introduction

Globally, the incidence of neonatal sepsis is estimated at approximately 1–50 per 1000 live births, widely varying between different countries and geographic regions; these discrepancies reflect the differences in health resources, maternal and neonatal risk factors, and prevention strategies, and they also depend on the time of disease onset [1,2]. Neonatal sepsis is classified into early-onset (EOS) and late-onset sepsis (LOS). EOS occurs within 72 h after birth, affecting 0.5–1 in 1000 infants in high-income countries, with a mortality rate of 10–15% [3,4]. LOS is characterized by onset of symptoms after 72 h of life due to exposure of neonates and infants to microorganisms of the community or hospital environment. Preterm neonates are considered the most vulnerable population, with reported rates of hospital LOS as high as 40% in extremely preterm neonates. In contrast, LOS due to community microorganisms mainly concerns late preterm and full-term infants [5,6]. Population estimates of confirmed neonatal sepsis in high-income countries (HICs) have been adequately studied and documented. Regarding low- and middle-income countries (LMICs), relative data are scarce and blurred [7]. Despite advances in perinatal care, neonatal sepsis remains one of the leading causes of neonatal mortality accounting for 3–30% of infant and childhood deaths with an estimated death toll of over 400,000 annually worldwide [1,8]. The World Health Organization (WHO) recently identified the need to reduce the burden of neonatal sepsis as the Sustainable Development Goal 3 aiming at the reduction of neonatal mortality to a maximum of 12 per 1000 livebirths by 2030 [9]. Still, this target appears to be unachievable without the significant reduction in sepsis-related neonatal deaths in LMICs [10].

The microorganisms mostly causing neonatal sepsis differ depending on the time of disease onset. In EOS, usually associated with vertical transmission of pathogens from mother to child, group B streptococcus (GBS), *Escherichia coli*, *Staphylococcus aureus*, *Coagulase negative staphylococci* (*CONS*), *Haemophilus influenzae*, and *Listeria monocytogenes* are reported as the most common pathogens. In LOS, which is mainly considered a nosocomial infection but could be a community infection as well, the predominant pathogens are *CONS*, *Staphylococcus aureus Escherichia coli*, *Klebsiella pneumoniae*, *Pseudomonas aeruginosa,* and GBS [1]. Following the guidelines of the American Academy of Pediatrics (AAP) and American College of Obstetricians and Gynecologists (ACOG) regarding universal screening by culture of all pregnant women from 35 to 37 weeks gestation and the use of intrapartum antibiotic prophylaxis (IAP) to prevent perinatal GBS to women with culture positive for GBS, a significant reduction in the incidence of EOS GBS-associated neonatal sepsis has been recorded worldwide [1,11].

The widespread use of antibiotics has resulted in a significant change in the etiological profile of neonatal sepsis over the years. In the 1930s and 1940s, group A streptococcus (GAS) was the main pathogen of neonatal sepsis, while nowadays it is rarely the cause of neonatal sepsis [12,13]. While the WHO reported an incidence of neonatal GAS bacteremia of 0.55 per 1000 live births in 2005 [14], there is a recent meta-analysis reporting a pooled incidence of neonatal invasive GAS disease (iGAS) worldwide of 0.04 (95% CI 0.03–0.05) per 1000 livebirths [15]. GAS, also known as *Streptococcus pyogenes*, is a beta-hemolytic bacterium that belongs to Lancefield serogroup A and it is a nonmotile, non-spore-forming coccus. There are many different serotypes of *S. pyogenes*, each of which is characterized by the production of a series of pyrogenic exotoxins that contributes to their ability to invade tissues and lead to the manifestation of systematic disease and septic shock [16,17]. GAS is a highly transmittable pathogen that can occasionally colonize carriers who remain asymptomatic or cause mild localized and self-limited infections, such as tonsillitis, scarlet fever, and Impetigo. However, iGAS can also cause rare yet severe cases of infectious conditions such as TSS, manifesting as an early onset of shock and systematic multiorgan failure, necrotizing fasciitis (NF) leading to local necrosis of the subcutaneous soft tissues and skin, and bacteremia that can subsequently cause secondary localized infections including meningitis, pneumonia, peritonitis, osteomyelitis, septic arthritis, myositis, surgical site infection, as well as postpartum sepsis, the latter being one of the main causes of maternal mortality [15,17]. The incidence of these invasive infections is very low, at about 1–8/100,000 per year. However, their potential mortality rates range between 5% and 20%, while in cases of septic shock, they can be as high as 30–45% [18]. With regards to the postpartum and perinatal period, it is worth mentioning that 1/50 cases of iGAS infection present in postpartum women, and in these cases, there is a mortality rate of 3.5%. The incidence of invasive neonatal infection caused by GAS has not been clearly defined and recorded. However, according to data from the United Kingdom, it is reported to be approximately 1.5/100,000 people per year, whereas in the United States of America, within the 7-year time period of 2009–2016, an increasing frequency of invasive GAS infections in children has been noted ranging between 0.16 and 0.37 for every 1000 hospital admissions [19,20].

In December 2022, the WHO reported an increased incidence of scarlet fever and cases of iGAS in Europe and the United States but with a low risk of iGAS for the general population [21]. However, during the same period, several public health agencies issued warnings about an unusually high number of iGAS infections, especially in the pediatric population [22,23]. Furthermore, in the UK, an alert was issued reporting an unusual increase in non-invasive GAS (mainly tonsillitis and scarlet fever) and iGAS with several deaths in children under ten (up to 24) in a short period of time [24,25]. Although the importance of GAS infection has been recognized in children and adults, especially in HICs, there is limited knowledge regarding the severity of iGAS in neonates, a potentially high-risk population, and the risk of later neurodevelopmental impairment [15]. Understanding the severity and the possibility of adverse outcomes of iGAS in the neonatal population is essential for the prevention and appropriate management of the disease. Therefore, we conducted a systematic review to identify the risk factors, the clinical presentation, and the outcome of GAS infection in neonates.

## 2. Materials and Methods

### 2.1. Search Protocol/Databases

We relied on systematic review methodology to identify, evaluate, and interpret available research that answered our research objective. For the present systematic review, a protocol was designed and written following the Preferred Reported Items for Systematic Reviews and Meta-analysis (PRISMA, presented as Supplementary Material) guidelines [26] and Meta-analysis (PRISMA, presented as Supplementary Material) guidelines

, which is registered in the PROSPERO database (PROSPERO 2023 CRD42023443067, available at: https://www.crd.york.ac.uk/prospero/display_record.php?ID=CRD42023443067, accessed on 6 July 2023). The systematic review of the literature was carried out during the period July 2023–September 2023. An electronic search of the existing literature was conducted in the online PubMed and Scopus databases until 3 September 2023 by using a combination of the following key words: “sepsis”, “septicaemia”, “septicemia”, “bacteremia”, “meningitis”, “invasive infection”, “necrotizing fasciitis”, “streptococcus pyogenes”, “group A streptococcus”, “group A streptococcal”, “neonatal”, “neonate”, “newborn”, “premature”, and “preterm” with Boolean logic operators.

Additionally, in order to reduce the risk of missing data and to fully cover the entire extent of the available literature, a manual electronic search and review of the references of each selected study, as well as references from previous systematic reviews related to our research field, was performed.

All observational studies (cohort studies, cross-sectional studies, case–control studies, case series, case reports) and clinical trials referring to GAS infection in the neonatal population were included. GAS infection was defined as positive blood, urine, CSF, or other sterile anatomical site affected in neonates. Furthermore, the cases with isolation of GAS from a non-sterile site in neonates with necrotizing fasciitis or streptococcal toxic shock syndrome (TSS) were also included. Only studies published in the English language were included and there were no geographical or chronological limitations.

### 2.2. Exclusion Criteria

- Studies that included neonates in the same group as children and adult patients and did not provide specific data regarding the neonatal population.
- Systematic reviews, meta-analyses, as well as studies coming from conference proceedings and those limited only to an abstract.

### 2.3. Study Outcome(s)

- Risk factors/characteristics of the affected neonates.
- Clinical presentation of GAS infection in neonates.
- Outcome of the neonates with GAS infection including mortality.

### 2.4. Data Synthesis and Presentation

Retrieved data were extracted into a Microsoft Excel template and they were recorded according to the following: infection site, time of disease onset, number of participants, other relevant study population grouping criteria (neonatal subpopulations: full-term neonates, preterm neonates, very low birth weight neonates), study design, year of publication, and other grouping criteria (such as maternal and perinatal factors) with the aim of pooling and meta-analyzing study results, where possible.

### 2.5. Disagreement Resolution

Screening, data extraction, and quality assessment were conducted independently by three investigators (AK, FF, VD), with conflicts resolved by discussion and consensus between them or, if necessary, by a fourth investigator (RS).

## 3. Results

### 3.1. Study Selection

**Figure 1:**
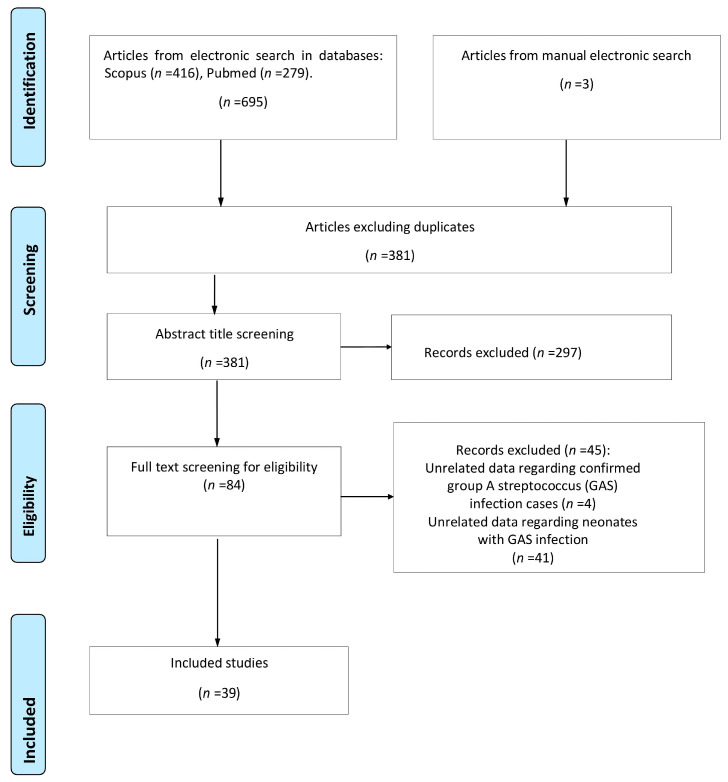
Flow chart of the systematic review study selection.

A total of 698 studies were retrieved from the search of the electronic databases. Of this total, 317 were duplicate records and were removed by a single researcher using EndNote X8. After screening the title and abstract of the remaining studies, 297 studies were excluded either because their subject matter did not serve the purpose of the study or because they met some of the exclusion criteria. Thorough reading of the full text of the remaining 84 studies revealed that only 39 studies met all the inclusion criteria and were included in this review. The flow chart is shown in Figure 1.

### 3.2. Study Characteristics

**Figure 2:**
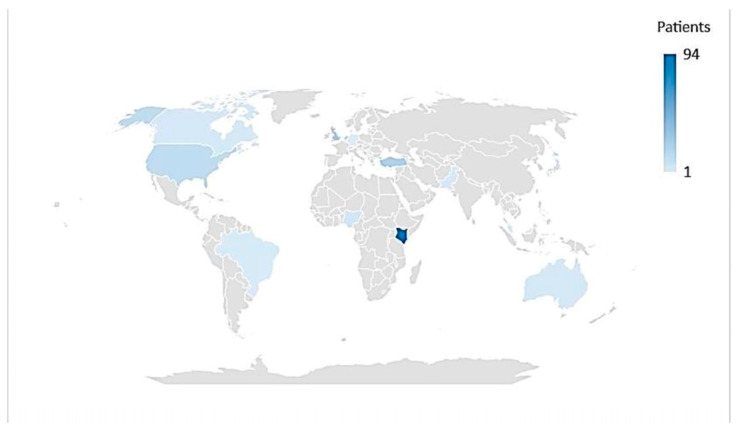
Countries with data on invasive group A streptococcal disease in neonatal population.

Among the studies included in this systematic review, 5 were observational, 16 were case series, and 18 were case reports. The countries from which the studies originate are the USA, Canada, Australia, Germany, Belgium, Brazil, Denmark, the UK, Kenya, Japan, Malaysia, Nigeria, Pakistan, Portugal, Serbia, Turkey, The Netherlands, and Israel (Figure 2).

Overall, these studies evaluated data from 194 neonates. Unfortunately, there were a lot of missing data among the retrieved studies. The demographic data of the study population are presented in Table 1.

**Figure 3:**
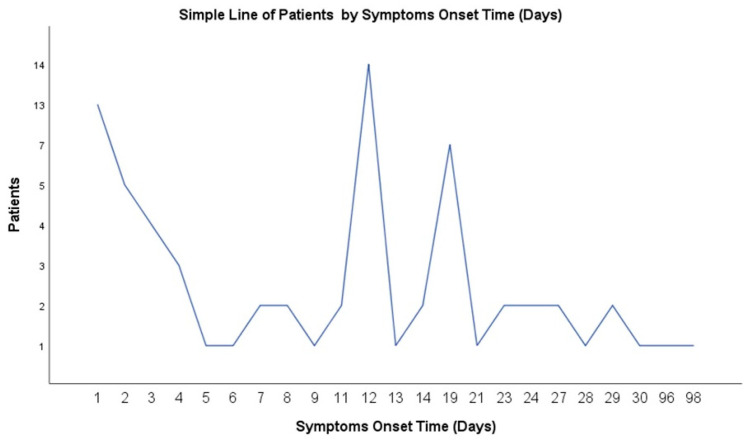
Distribution of the number of neonates that presented with invasive GAS disease according to the time of disease onset (days of life). Total of 69 neonates.

According to reported data, preterm neonates constituted the majority of the study population (30/43, 69.8%); a predominance of vaginal deliveries was recorded. Regarding the time of disease onset, data are given only for 69 (35.5%) cases (Figure 3), with EOS presenting in 20 (30%) and LOS in 49 (71%) neonates (analytical data are presented in Supplementary Tables S1 and S2, respectively), while for 125 iGAS cases, no information was given regarding the time of disease onset (Supplementary Table S3).

From our review, we concluded that the clinical presentation in the study population varied depending on the time of disease onset. Thus, in neonates with EOS, the primary symptoms were respiratory distress (60%), while in neonates with LOS, the disease was mainly presented with rash (46.9%), fever (30.6%), or gastrointestinal disturbances (44.9%) (Table 2).

As far as it concerns the isolation site of the pathogen, from our review, it emerged that GAS was isolated from blood cultures in 150/194 (77.3%) cases, skin lesions in 65/194 (33.5%), pleural fluid in 5/194 (2.6%) cases, and cerebrospinal fluid in 10/194 (5.2%) cases. Data on vaginal culture of mothers during pregnancy are available for 16 women, with 12 of them being GAS-positive; 12 offspring presented with EOS. Vertical transmission was documented in 16/17 (94%) EOS disease cases. Omphalitis (9.3%) and meningitis (5.2%) were identified as the predominant clinical expressions of disease; interestingly, five cases with TSS were also reported (Table 3).

Treatment management was not reported in 129 retrieved cases. In most cases, a combination of two or three antibiotics was used that mainly included cephalosporin (50.8%), penicillin (26.3%), and ampicillin (26.2%). Only 14 neonates were treated with penicillin as monotherapy; surgical treatment was needed in two cases. Notably, one case reported from Pakistan referred to a septic neonate whose parents denied treatment and the outcome was still good. The duration of antibiotic therapy was 11–28 days.

The outcome was good for the majority of neonates with a mortality rate of 13.8% (26/189 cases with data available). The mortality rate in neonates with EOS was 30% (6/20), while in those with LOS, a lower mortality rate of 11.1% (5/45) was recorded. From our review, we could not draw any conclusions regarding the GAS serotypes’ identification and disease clinical manifestation or patients’ outcome because the data collected from the various studies were limited and characterized by a large heterogeneity in terms of the method used and cases tested for serotyping.

## 4. Discussion

A small amount of data are available in the literature regarding iGAS infection in neonates, while the potential severity of the disease calls for prompt recognition and diagnosis and proper treatment. In order to collect all the existing data and update our knowledge and practices with regards to the risk factors, the clinical presentation, and the outcome of GAS infection in the neonatal population, we performed a systematic review of the available bibliography.

Neonatal infections are commonly caused by pathogens such as GBS, Escherichia coli, CONS, Haemophilus influenzae, Listeria monocytogenes, Staphylococcus aureus, Klebsiella pneumoniae, and Pseudomonas aeruginosa [1]. Group A streptococcus is a rather uncommon causal factor that can, however, lead to an invasive infection with a potentially fatal outcome [27]. The iGAS disease can be defined as the isolation of GAS in normally sterile places such as blood, CSF, joints, and pleural and peritoneal fluid.

Based on the time point of onset of symptoms, iGAS infections in neonates are characterized as early-onset (EOS) and late-onset (LOS) sepsis. Nevertheless, the clinical characteristics of each group are not well defined. Our systematic review highlighted the presence of differences with regards to clinical presentation, infection sites, and outcomes of GAS invasive disease between neonates with EOS or LOS and this finding is in accordance with the results of a previous systematic review [13]. Besides primary septicemia, the main clinical expressions of EOS infection appeared to be pneumonia, empyema, soft tissue infection, and TSS. Common characteristics of EOS included respiratory distress, rapid deterioration, and high mortality rate irrespective of the infection site. Approximately 30% of the neonates presented with a rash upon disease onset. Fever was present in approximately 30% of the neonates with EOS infection as opposed to the findings of Miyairi et al. [13], where the absence of fever and neutropenia appeared to be a common finding, suggesting the variability of response of neonates to GAS infection. On the other hand, the main types of clinical presentation of late-onset disease in the population of our systematic review, besides primary septicemia, were peritonitis and meningitis, while pneumonia and TSS were not noted in any case. As opposed to symptoms of early-onset disease, rash, gastrointestinal tract symptoms, and fever appeared to be the most frequent symptoms/clinical signs and manifestations of LOS disease and these findings were similar to the ones of a previous study [13]. In our systematic review, 65% of the neonates that were included could not be classified as presenting with EOS or LOS disease because the studies referring to them did not define the time of disease onset. In our study’s general population, except for primary septicemia, meningitis, omphalitis, and peritonitis were included in the main types of clinical expressions of iGAS disease. Furthermore, the mortality rate that we recorded for iGAS disease in all neonates (approximately 14%) can be considered far from insignificant. With regards to the clinical presentation, it is worth mentioning that nine of the recorded cases of iGAS disease presented as primary peritonitis. Peritonitis is defined as the inflammation of the peritoneum and it is usually described as primary, secondary, or catheter-related. Primary peritonitis constitutes a rare entity among the healthy pediatric population [28]. Neonatal peritonitis may have a bacterial or chemical cause. The majority of cases of bacterial peritonitis in this age group can be attributed to Necrotizing Enterocolitis (NEC), complications of a bowel obstruction, or iatrogenic stomach or duodenum perforation [29]. Primary peritonitis remains a rare entity in the pediatric population and group A streptococcus (GAS) is a rather unusual cause [28]. The pathophysiology of primary peritonitis remains to be revealed, although several mechanisms have been suggested for the bacterial spread such as hematogenous, lymphatic, ascending inoculation from the urogenital system and bacterial translocation of the gastrointestinal tract flora [30]. In the adult population, ascending inoculation through the urogenital system has been suggested to cause primary peritonitis from GAS [31,32].

Several virulence factors contribute to the pathogenic complexity of GAS. One of the most significant is protein M which is encoded by the gene emm. Until today, more than 200 types and subtypes of emm variations have been traced, depending on the geographical locus [33]. A strong correlation has been reported between the strains causing rheumatic fever and category I M protein [16]. Besides the M protein, there are more particles of the streptococcus surface that are involved in the host immune response [16]. More than 100 different types of protein M have been identified so far; some of them are known to correlate with specific manifestations of the disease. In our systematic review, we were not able to draw conclusions regarding the above statement because the data we collected from the several studies included were limited and highly heterogenic with regards to the methods and the cases that underwent serotyping. In a previous review that was looking into the iGAS infections in the neonatal population, M1 and M3 were found to be the dominant serotypes, representing approximately 68% of the streptococcus specimens that underwent serotyping, while there was a significant correlation noted between serotype M1 and early onset of the disease. The correlation between M1 and M3 serotypes and invasive GAS disease has also been reported in studies of children and adult populations [34,35].

Based on the systematic review of the bibliography, the management of severe invasive iGAS disease consists mainly of specific antimicrobial treatment as well as supportive care with fluids and electrolyte supplementation, minimizing or counteracting the effects of toxins and supplemental practices based on specific cases, for example, excessive surgical drainage that may be necessary in cases of deeper abscesses in order to control and remove the infection source. Penicillin remains the treatment of choice because there has been no report of resistance to this antibiotic. The addition of clindamycin in the treatment regime can prove beneficial especially in cases of Streptococcal Toxic Shock, because it can suppress the production of exotoxins and proteins from GAS. Moreover, clindamycin has a longer half-life and does not act antagonistically to penicillin [19,36].

In the beginning of the 20th century, GAS was one of the leading causes of neonatal and maternal mortality, but the use of antimicrobial medicine has drastically reduced the incidence of neonatal GAS infection. However, in the mid-1980s, a resurgence of group A streptococcus infections was recorded in the neonatal population despite the use of penicillin and the fact that streptococcus remains sensitive to it. According to many researchers, the high percentage of neonatal cases that were recorded during that time period could be related to an increasing trend of the invasive disease cases that had already been noted in children and adults [13,37,38]. An increase in the number of cases of invasive disease from group A streptococcus was also recorded in some European countries in children younger than 10 years old during 2022, especially from September onwards [22,23]. In both France and the United Kingdom, the incidence of iGAS disease in children was markedly higher compared to the same time period in the pre-COVID-19 era. During the same time period, many fatal cases of iGAS in children younger than 10 years old were also reported [24,25,39]. Whether this increase in the number of group A streptococcus invasive disease cases in children will have an impact on the incidence of vaginal mucosa colonization in women of reproductive age and subsequently neonatal iGAS disease cannot be clearly answered as yet.

According to our systematic review, despite the limited available data with regards to the means of transmission, vertical transmission appeared to account for the majority of invasive early-onset GAS disease cases, while the means of transmission was not identified in the majority of late-onset invasive GAS disease cases. These findings could indicate that the late disease onset is a manifestation of a relatively low bacterial load obtained through vertical transmission or a postnatal localized GAS infection, and this is in concordance with findings from other studies as well [13]. It is worth mentioning that, despite the limited data, maternal complications also appeared to be more frequent in the group of EOS disease neonates because there was a high percentage of postpartum sepsis or TSS, and this finding had also been recorded by Miyairi et al. [13]. With regards to the vaginal smear cultures during pregnancy, there were data from only 16 mothers, 12 of which were positive and were taken from mothers whose neonates presented with EOS infection. Approximately 30% of the neonates in the EOS group were born preterm, and this might have been to some extent the result of GAS-related chorioamnionitis. Vertical transmission was documented in 94% of EOS disease cases and this percentage is significantly higher than the one that was described in a previous systematic review [13]. The overwhelming nature and presentation of the disease and the simultaneous maternal factors indicate that the early onset of the neonatal GAS disease could be a manifestation of hematogenous dispersion or of a clinical syndrome caused by the exposure of the fetus to the microbial toxins while in utero. This suggestion is further supported by data documenting a high rate of fetal mortality among the cases of maternal iGAS disease with a prenatal onset [40,41,42]. The findings from our review are similar to the findings of the review run by Miyairi et al. [13] and highlight the crucial role of vertical transmission in the pathophysiology of the disease and the invasive nature of GAS disease, especially in cases of early perinatal infection. This resembles the cases of neonatal GBS infection where maternal testing and prophylaxis during labor have led to a significant decrease in the disease frequency [43,44].

There are limited data in the literature regarding the administration of antibiotic prophylaxis for the prevention of GAS disease both in neonates and their mothers. In a population-based surveillance survey for postpartum GAS disease, it was suggested that the postpartum maternal disease can be prevented [42,45]. At present, there is no recommendation for screening of asymptomatic women during pregnancy. However, given the exceptional risk for both the pregnant woman and the neonate during this period, there is a need for more studies regarding the necessity, the means, and the cost analysis of presymptomatic testing [17]. Cases of women presenting with postpartum fever and/or abdominal pain call for a high suspicion of GAS; therefore, investigation with blood cultures and vaginal smears should be taken into consideration and is recommended according to the most recent directive of the International Society for Infectious Disease in Obstetrics and Gynecology (ISIDOG) [19]. Given the potential of severe disease and the rapid deterioration, the development of quick analysis methods for the detection of GAS in vaginal smears could allow for prompt diagnosis and administration of the indicated antibiotic treatment. There is a need for further studies about the necessity and the most appropriate method of testing and treatment. Positive swab results should be interpreted properly and lead to prompt treatment, given the probability for severe maternal and neonatal disease and complications [45]. Many of the health care professionals who are looking after pregnant women, including obstetricians and midwives, have very limited experience in the recognition and/or management of these conditions. Therefore, it is very important to raise awareness and create easily accessible guidelines that could facilitate the prevention and management of not only maternal but also neonatal severe iGAS disease. Prompt recognition and action against this rare but potentially lethal disease could be some of the many characteristics of these guidelines. The International Society for Infectious Diseases in Obstetrics and Gynecology (ISIDOG) provides every health care professional engaged with the care of women with a scientific forum and offers recommendations for less-frequent infectious diseases that are often not included in the guidelines of other scientific societies.

The pathophysiology of LOS GAS disease is not well understood, and the infection does not always appear to be related with perinatal parameters. Transmission in the community by an asymptomatic carrier, that usually is the baby’s mother, should always be taken into consideration and every roommate should be tested with a rapid antigen detection test for GAS in the oropharyngeal secretions. The prevalence of GAS carriers among children is 12% and varies with age [46,47]. In a recent study, Germont et al. [27] described the clinical and laboratory characteristics and outcomes of iGAS infection in infants aged less than 3 months that were hospitalized between 2007 and 2016 in France, and in this study, 73.7% of the cases were found to be attributed to GAS community contacts, while in case of neonates, the respective percentage was up to 88.9%. This finding was in accordance with a study of the evaluation of GAS transmission in households in England; the authors described that the risk of iGAS transmission significantly increased within the household after a single case, especially in the groups of mothers and infants during the neonatal period (RR: 11.9, C.I. 95%: 2.0–70.3). In this particular population, the theoretical number of cases that would demand to be treated in order to prevent one secondary case by the use of antibiotic prophylaxis was 50 as opposed to 271 for the general population [48]. Presymptomatic testing and treatment of carriers is recommended in specific cases such as outbreaks of invasive GAS disease [46]. Even though not consistently applied, in some countries such as the United Kingdom and Ireland, prophylaxis is recommended for the duo of mother–neonate when either of the two develops iGAS disease [48]. The high rate of community-acquired resistant sepsis constitutes a severe problem of global public health, thus supporting the use of cefotaxime and ampicillin as empirical treatment regimens [49]. However, in cases of infections caused by community strains of GAS, benzyl-penicillin remains the antibiotic treatment of choice because there has been no resistance described. Alternatively, ampicillin can be used as all of the reported community strains of GAS are sensitive to both of the antibiotics. GAS has a high rate of resistance to gentamycin and resistance to erythromycin is also reported to be increasing in certain countries. As far as clindamycin is concerned, the resistant strains are mainly related to multi-drug resistant bacterial clones [50,51,52].

Furthermore, group A streptococcus infections in the pediatric population have recently been correlated with neurodevelopmental impairment [41,53,54]. Unfortunately, the majority of studies that fulfilled the inclusion criteria of our systematic review did not refer to the long-term follow-up of the neonates with iGAS disease so as to facilitate drawing inferences about the neurodevelopmental impairment during infancy and childhood.

It must be noted that our study does have some limitations and its findings should be interpreted with caution. The main limitation was that our study was based solely on data that were collected basically from case reports and case series studies, which are known to be characterized by a limited capability of generalization and validity as well as the inability to form cause–result links. Moreover, there might be significant bias related to publication, the retrospective design of the study, and the focus on rare or unusual cases. On the other hand, it has been noted and suggested that some case reports/studies and case series can potentially carry relevant knowledge that should be taken into consideration when composing a systematic review in the occasion where there are no available data from RCTs and observational studies, especially when even a small number of studies report a significant and probable causal relation in the cases of an epidemic or a side effect of a medicine that has recently entered circulation [55,56,57]. The gaps or the significant heterogeneity in the recording of fundamental information, such as the time point of the disease manifestation, the existence of prenatal risk factors, the demographic characteristics of the neonates, means of disease transmission, as well as the method of diagnosis, among the studies that were included in our review are apparent and these were the main inhibitory factors in performing a meta-analysis.

## 5. Conclusions

In conclusion, neonatal GAS disease can be manifested as an early- or late-onset disease characterized by a high rate of neonatal morbidity and mortality. Even though iGAS disease constitutes a rare infectious entity in the neonatal population, the potential severity of the disease calls for prompt recognition and appropriate treatment. In the majority cases, the critical point that is related to the septic mortality of the patient is the delay in the diagnosis that subsequently leads to a delayed therapeutic management. Because there are no randomized trials looking into the invasive neonatal GAS disease, we believe that our systematic review, that is mainly based on data from case reports, could potentially be an efficient and very useful way of informing and raising awareness among the health care providers regarding the prevention and management of neonatal iGAS cases, through the discussion of risk factors, disease clinical manifestations, and the conditions that are related to the outcome of the neonates. The high rate of vertical transmission and the maternal complications as well as the very high incidence of community-acquired neonatal GAS infections indicate the necessity to evaluate if systematic antibiotic prophylaxis and close observation can facilitate avoiding these severe infectious cases. Last but not least, the potential of extending the prenatal screening with regards to GAS and the possibility of prophylaxis administration during labor must be considered and studied.

## Figures and Tables

**Table 1:** Demographic data of the study population.

		Reported Data	Missing Data
Gestational age (weeks)	Preterm	30/43 (69.8)	151/194 (77.8)
	Term	13/43 (30.2)	
Gender	Male	35/59 (59.3)	135/194 (69.6)
	Female	24/59 (40.7)	
Delivery mode	Vaginal	17/26 (65.4)	168/194 (86.6)
	Cesarean section	9/26 (34.6)	

Data are presented as absolute frequencies (percentages) when appropriate.

**Table 2:** Clinical presentation of study population.

	EOS	LOS
Respiratory distress	12/20 (60)	7/49 (14.2)
Fever	6/20 (30)	15/49 (30.6)
Rash	6/19 (30)	23/49 (46.9)
Gastrointestinal disturbances	4/20 (20)	22/49 (44.9)

Data are presented as absolute frequencies (percentages) when appropriate.

**Table 3:** Information on the foci of infection of study neonates.

	All Cases (*n* = 194)	EOS (*n* = 20)	LOS (*n* = 49)
Meningitis	10	-	5
Pneumonia (empyema)	5 (4)	5 (3)	-
Omphalitis	18	-	-
Peritonitis	9	-	8
Skin lesions	5	-	1
Osteomyelitis/arthritis	2	-	2
TSS	5	4	1

## Data Availability

Data are contained within the article.

## Supplementary Materials

The following supporting information can be downloaded at https://www.mdpi.com/article/10.3390/jcm12226974/s1, PRISMA checklist and Supplementary Tables S1–S3 [13,19,37,38,42,46,47,52,58,59,60,61,62,63,64,65,66,67,68,69,70,71,72,73,74,75,76,77,78,79,80,81,82,83,84,85,86,87,88]. Table S1: Studies included EOS neonatal cases with of iGAS; Table S2: Studies included LOS neonatal cases with of iGAS; Table S3. Studies included neonatal cases of iGAS with no data regarding the time of disease onset.
