# Supplementary material for: Group A Streptococcus Infection in Neonatal Population: A Systematic Review of The Literature
Source: J Clin Med. 2023 Nov 8;12(22):6974. doi: 10.3390/jcm12226974 (PMC10672068; doi:10.3390/jcm12226974)
Supplement: Supplementary file 1 [file jcm-12-06974-s001.zip › jcm-2655405-supplementary-Tables S1-S3.pdf]

Supplementary Table S1. Studies included EOS neonatal cases with of iGAS.

First author, publication year	Study type	Patients (N)	Gestational Age (weeks)	Birth weight (g)	Origin	Gender	Mode of Delivery	Length of Rupture of Membranes (hours)	Maternal symptoms	Maternal GAS positive Cultures	Neonatal positive cultures	Maternal Peripartum Antibiotics	Clinical manifestations	Diagnosis	Microorganism(Strep A emm strains)	Complications	Type of treatment	Treatmnt duration (days)	Neonatal outcome
Middleton, 2014 [1]		1	33	1910	Netherlands	Male	N/R	N/R	None	N/R	Skin, umbilicus, ear, blood, pleural fluid	N/R	Respiratory distress, hypotension	Sepsis, pneumonia	N/R	N/R	Penicillin	N/R	Death
Crum, 2002 [2]	Case report	1	34	2216	USA	N/R	Caesarean section	0	Toxic shock syndrome (TSS)	Vaginal	N/A	gentamicin, clindamycin	Cardiorespiratory failure	respiratory distress	N/R	Stage III hypoxic brain damage due to asphyxia at delivery, grade IV intraventricular hemorrhage, hydrocephalus, and brainstem herniation	Ampicillin and cefotaxime	N/R	Death
Mahieu, 1995 [3]	Case report	1	35	N/R	Belgium	Male	Vaginal	19	Tenderness of uterus	Placenta, umbilical	Blood, bullae	Penicillin	Grunting, apnea, decreased feed-	TSS	M1, T1, SOR-	Skin necrosis and formation of bullae,	Ampicillin, netilmycin → penicillin	21 days	Good

										cord, cervix, and amniotic membranes			ing, bradycardia, oliguria, hypotension, respiratory distress			cranial cellulitis, superior vena cava obstruction			
Nieburg, 1975 [4]	Case series	1	36	2155	USA	Female	N/R	4,5	Leucorrhea	Blood, and lochia	Blood, urine	N/R	Respiratory distress, pulmonary hemorrhage, circulatory collapse, bilateral adrenal hemorrhage	sepsis	emm type 12	N/R	N/R	N/R	Death
		1	36	2600	USA	Male	N/R	4,5	Fever, pelvic thrombophlebitis	Blood	Blood	N/R	Respiratory distress, pulmonary hemorrhage, circulatory collapse, bilateral adrenal hemorrhage	sepsis	emm type 12	N/R	N/R	N/R	Death
		1	38	3755	Netherlands	Female	N/R	N/R	Puerperal sepsis	N/R	Skin, ear, eye, umbilicus, nasopharynx	N/R	Fever, conjunctivitis, respiratory distress, hypotension	Sepsis, pneumonia	N/R	N/R	Penicillin	N/R	Good
Lequier 1998 [5]	Case series	1	38	2480	USA	Female	N/R	N/R	N/R	Vaginal and blood	Blood	N/R	Pale and lethargic	sepsis, empyema	emm type 1	Pleural empyema	Penicillin, empyema decortication	N/R	Good

		1	39	3775	Netherlands	Male	N/R	N/R	TSLS	N/R	Skin, nasopharynx, umbilicus, blood	N/R	Respiratory distress, hypotension, skin rash	toxic shock-like syndrome (TSLS)	N/R	N/R	Penicillin, clindamycin	N/R	Good
Shinoda, 2020 [6]	Case series	1	39	3158	Japan	Female	Vaginal	N/R	N/R	blood	Blood, skin swabs, and cerebrospinal fluid	N/R	Fever	sepsis, meningitis	emm 80	N/R	Ampicillin, cefotaxime, and acyclovir	14 days	Good
		1	39	N/R	UK	Male	Caesarean section	N/R	Fever	Uterine cavity, placenta	Blood, umbilicus	N/R	Drowsy, jaundiced, fever, abdominal erythema and distension	sepsis	N/R	N/R	N/R	N/R	Good
Verboon-Maciolek, 2000 [7]		1	40	3150	Netherlands	Male	N/R	N/R	Pharyngitis	N/R	Umbilicus, ascites fluid	N/R	Poor feeding, vomiting, distended abdomen, bloody stools	Peritonitis	N/R	N/R	Penicillin, laparotomy	N/R	Good
		1	40	3680	Netherlands	Male	N/R	N/R	Puerperal sepsis	N/R	Skin, ear, umbilicus, nasopharynx, blood	N/R	Fever, skin rash, respiratory distress, hypotension	TSLS	N/R	N/R	Penicillin, clindamycin	N/R	Good
Nohara, 2014 [8]	Case series	1	40	3144	Japan	Female	Vaginal	N/R	N/R	Vaginal	Throat, skin, head empyesis, pleural fluid	N/R	Small empyesis on the head, respiratory distress	pleural empyema	emm type 6	N/R	Ampicillin and cefotaxime	11	Good
		1	40	N/R	UK	Male	Vaginal	N/R	Sepsis	N/R	Blood, scalp wound swab,	N/R	Infected scalp wound,	sepsis	N/R	N/R	N/R	N/R	Good

											umbilicus, nose, throat		cellulitis, oedema, generalised rash						
Miyairi, 2004 [9]	Case report	1	term	4800	USA	N/R	Vaginal	12	N/R	N/R	blood	N/R	Cardiorespiratory arrest	sepsis	N/R	N/R	Ampicillin , gentamicin	N/R	Death
Nutman, 1979 [10]	Case report	1	term	2700	Israel	Female	Vaginal	14	N/R	cervix	Nose, skin, CSF, blood	N/R	Erythematous lesion was observed on the right cheek, poor feeding, fever	sepsis, meningitis	type M52	N/R	Penicillin, gentamicin, and chloramphenicol→ penicillin	N/R	Good
Thaarup, 1997 [11]	Case report	1	term	3325	Denmark	Female	N/R	30 min	fever	blood, cervix and an infected perineal rupture	Pleural fluid	N/R	Tachypnea, costal retractions, grunting and peripheral cyanosis	Pleural empyema	N/R	N/R	Ampicillin and netilmicin → Penicillin and netilmicin → ceftriaxone and netilmicin	28 days	Good
Ross, 2021 [12]	Case report	1	N/R	N/R	Australia	N/R	Vaginal	N/R	Abdominal pain	Vaginal, breast milk	Blood, scakp wound	N/R	Tachypnoea and grunting, subgaleal haemorrhage, haemodynamic instability and fever	Sepsis	N/R	N/R	Ampicillin, gentamicin and cefotaxime → ampicillin	14 days	Good
Sakata, 2013, 2014 [13,14]	Case series	1	N/R	N/R	Japan	Female	N/R	N/R		N/R	Pleural fluid	N/R	N/R	Pyothorax	emm type 6	N/R	Ampicillin	N/R	Good
Timmis 2010 [15]	Case series	1	N/R	N/R	UK	Male	N/R	N/R		N/R	Blood	N/R	N/R	Definite streptococcal toxic shock	N/R	N/R	Cefotaxime gentamicin benzylpenicillin	N/R	Death

													syndrom (DSTSS)					

Supplementary Table S2. Studies included LOS neonatal cases with of iGAS.

First author, publication year	Study type	Patients (N)	Gestational Age (weeks)	Birth weight (g)	Origin	Gender	Mode of Delivery	Length of Rupture of Membranes (hours)	Maternal symptoms	Maternal GAS positive Cultures	Neonatal positive cultures	Clinical manifestations	Diagnosis	Microorganism(Strep A emm strains)	Complications	Type of treatment	Treatment duration (days)	Neonatal outcome
Middleton, 2014 [1]	Case series	1	30	N/R	Australia	Female	N/R	N/R	N/R	N/R	Peritoneal Fluid	Hypothermia, tachycardia, tachypnoea, grunting, hypoxia, hypotension, cool peripheries, sunken fontanelle, abdominal distension, bruish	TSLS	N/R	N/R	Ceftriaxone, gentamicin, metronidazole → piperacillin-tazobactam, exploratory laparotomy, surgical debridement of the area of necrosis	14	Good
		1	30	N/R	Australia	Female	N/R	N/R	N/R	N/R	Blood	Cough, lethargy, decreased	Sepsis	N/R	N/R	Ceftriaxone and flucloxacillin → piperacillin-	14	Good

												oral intake, hypothermia, hypoxia, poor peripheral perfusion, increased tissue turgor, sunken fontanelle, hypotension				tazobactam, vancomycin, azithromycin and oseltamivir→ benzylpenicillin		
Barnham 2001 [16]	Case series	1	35	N/R	UK	Male	Vaginal	27 hours	N/R	N/R	Blood	Dusky, floppy, lethargy	sepsis	N/R	N/R	N/R	N/R	Good
		1	36	2550	Netherlands	Male	N/R	N/R	None	N/R	CSF, brain abscess	Fever, poor feeding, full anterior fontanelle, seizures	Meningitis, brain abscess	N/R	N/R	Penicillin	N/R	Death
Ventura 2019 [17]	Case report	1	36	N/R	Portugal	Female	Vaginal	3 hours	N/R	N/R	Blood	Feeding refusal and hyporeactivity, dry mucous membranes, depressed anterior	Sepsis	emm type STG7882	N/R	Cefotaxime, ampicillin,	N/R	Good

												fontanelle, and weight loss						
Saito, 2017 [18]	Case report	1	37	N/R	UK	Male	Vaginal	N/R	N/R	N/R	Blood	Poor feeding, irritability, vomiting, loose stool and fever, erythema in umbilicus, tachypnea, tachycardia	Sepsis	emm st28.0	N/R	Cefotaxime and amoxicillin	14 days	Good
Huber, 2014 [19]	Case report	1	39	N/R	Germany	Male	Caesarean section	N/R	N/R	N/R	Blood	Fever, lethargy and poor feeding,irritability, palor and mottling	sepsis	N/R	Cellulitis-Adenitis	Ampicillin and gentamicin	14 days	Good
Verboon-Maciolek, 2000 [7]	Case series	7	40	3350	Netherlands	Male	N/R	N/R	None	N/R	Umbilicus, ascites fluid	Poor feeding, vomiting,distended abdomen, omphalitis	Peritonitis	N/R	N/R	Penicillin, peritoneal puncture	N/R	Good
Bhutta, 1990 [20]	Case series	1	40	3000	Pakistan	Male	N/R	N/R	N/R	N/R	Blood	Fever, poor feeding,	sepsis	N/R	Cardiorespiratory arrest	Cefotaxime,amikacin, cloxacillin	N/R	Death

												respiratory difficulty						
		1	N/R	N/R	Pakistan	Female	N/R	N/R	N/R	N/R	Blood and pus from the lesions	Lethargy, 2 vesicular lesions in the left axilla and antecubital fossa	sepsis	N/R	N/R	No treatment	N/R	Good
		1	term	2800	Pakistan	Male	N/R	N/R	N/R	N/R	Blood	Icteric, periorbital oedema, vomiting, necrotic umbilicus	sepsis	N/R	Cardiorespiratory arrest	Cefotaxime,amikacin, cloxacillin	N/R	Death
Campbell, 1996 [21]	Retrospective cohort	1	26	920	USA	N/R	N/R	N/R	N/R	N/R	Blood and throat	Apnea and poor perfusion	Sepsis	N/R	N/R	Benzathinepenicillin G intramuscularly, intravenous clindamycin	14	Good
		1	28	560	USA	N/R	N/R	N/R	N/R	N/R	Blood	Apnea, bradycardia, hypotension, and toxic shock-like syndrome, necrotic scalp lesion	Sepsis	N/R	Seven days later, he had apnea, shock-neutropenia, disseminated intra-	14-day course of penicillin G (200,000 units/kg per day) and also received a single dose of benzathine penicillin G	14	Death

															vascular coagulopathy, and,necrotizing cellulitis of the right ear and scalp extending to the occiput.	(50,000 units/kg)		
Wilschanski, 1989 [22]	Case report	1	term	N/R	Israel	Male	Vaginal	N/R	N/R	N/R	Blood	Fever, poor feeding, erythematous rash in right cervix + periorbital edema, focal seizure	Sepsis	3/13/B3264	N/R	Gentamicin, cefuroxime and ampicillin → Penicillin G	12 days	Good
Cartwright, 1977 [23]	Case report	1	term	4080	England	Male	Vaginal	N/R	N/R	N/R	Blood	Erythematous lesion, swollen cheek, oedematous eye	Sepsis	N/R	N/R	Penicillin, hydrocortisone	14	Good

Barnham, 1989 [24]	cohort study	1	preterm	N/R	UK	Male	N/R	Prolonged	N/R	N/R	Blood	Jaundice, sepsis	Sepsis	N/R	N/R	N/R	N/R	Good
Umadevi, 2013 [25]	Case report	1	term	3200	Netherlands	Male	Caesarean section	N/R	N/R	N/R	Arthritic fluid	Swelling on his right elbow and reduced movements of the right forearm	septic arthritis, sepsis	N/R	N/R	Ampicillin, cloxacillin and amikacin → Oral ampicillin and erythromycin	10 days	Good
Coulter, 1984 [26]	Case series	1	term	N/R	UK	Female	N/R	N/R	N/R	N/R	Blood, CSF, and umbilicus	Snuffles, irritability, poor feeding, bleeding from the tongue, and a transient rash on the face and trunk.	sepsis, meningitis	N/R	N/R	Penicillin, cefuroxime	N/R	Death
		1	term	N/R	UK	Male	N/R	N/R	N/R	Vaginal swab	CSF, paronychiae, umbilicus, nose, and nasogastric aspirate	Fever, opisthotonous, seizures, fontanelle distension.	meningitis	N/R	Hydrocephalus	Gentamicin and cefuroxime → penicillin	N/R	Good
Martic, 2010 [27]	Case report	1	N/R	N/R	Serbia	Male	N/R	N/R	superficial pyoderma	Skin	Blood	Fever , lethargy, poor feeding, erythema	sepsis	emm type 53.2	N/R	Vancomycin, amikacin → penicillin G	N/R	Good

Sakata, 2013, 2014 [13,14]	Case series	1	N/R	N/R	Japan	Female	N/R	N/R		N/R	Blood	N/R	Sepsis	emm type 1	N/R	Ampicillin	N/R	Good
		1	N/R	N/R	Japan	Male	N/R	N/R		N/R	Blood	N/R	Sepsis	emm type 1	N/R	Ampicillin	N/R	Good
		1	N/R	N/R	Japan	Female	N/R	N/R		N/R	Blood	N/R	sepsis , necrot-isizing fascilitis	N/R	N/R	N/R	N/R	N/R
Santos, 2009 [28]	Case series	1	N/R	N/R	Brazil	Female	N/R	N/R		N/R	CSF	N/R	meningi-tis	emm type 39,1	N/R	N/R	N/R	N/R
		1	N/R	N/R	Brazil	Female	N/R	N/R		N/R	CSF	N/R	meningi-tis	emm type 92	N/R	N/R	N/R	N/R
Shanks, 1986 [29]	Case re-port	1	N/R	N/R	USA	Male	Cae-sarean sec-tion	N/R	Fever, chills, vaginal hemor-rhage	Vaginal	Tunica vaginalis pus	Irritabil-ity, scro-tal swell-ing, fe-ver, yel-low exu-date of umbilicus	hydrocele infection	N/R	N/R	Nafcillin, gentamycin	N/R	Good
Torres-Martinez 1992 [30]	Case series	1	N/R	N/R	UK	Female	N/R	N/R		N/R	Blood, peritoneal fluid	Fever, rash, ab-dominal disten-sion, hy-poten-sion, shock	Peritoni-tis, sepsis	N/R	N/R	Penicillin	14 days	Good
Givner, 1991 [31]	Case series	1	N/R	N/R	Can-ada	Female	N/R	N/R		N/R	Blood	N/R	Sepsis, osteomy-elitis	N/R	N/R	N/R	N/R	N/R
Johnson, 1990 [32]	Case re-port	1	N/R	N/R	USA	N/R	N/R	N/R		N/R	Blood	N/R	sepsis	N/R	N/R	N/R	N/R	Good
Lehtonen, 1987 [33]	Case series	1	N/R	N/R	Tur-key	Female	N/R	N/R		N/R	Blood	Febrile , irritable, lethargic,	sepsis	N/R	N/R	Metronida-zole-cepha-lothin	N/R	Good

												abdomen distension and rash						
		1	N/R	N/R	Turkey	Female	N/R	N/R		N/R	Blood	Fever,irritable, lethargic, abdomen distension and rash	sepsis	N/R	N/R	Metronidazole-cephalothin	N/R	Good
Leonard, 2019 [34]	Retrospective study	12	N/R	N/R	UK	N/R	4 caesarean section,3 vaginal delivery, 5 N/R	N/R		N/R	Blood	5 fever, 2 respiratory distress , 2 infections of the umbilicus or ear, 1 hypoxic ischaemic encephalopathy, 2 N/R	sepsis	N/R	N/R	N/R	N/R	Good

Supplementary Table S3. Studies included neonatal cases of iGAS with no data regarding the time of disease onset.

First author, publication year	Study type	Patients (N)	Gestational Age (weeks)	Birth weight (g)	Origin	Gender	Mode of Delivery	Length of Rupture of Membranes (hours)	Maternal symptoms	Maternal GAS positive Cultures	Neonatal positive cultures	Maternal peripartum antibiotics	Clinical manifestations	Diagnosis	Microorganism(Strep A emm strains)	Complications	Type of treatment	Ttreatment duration (days)	Neonatal Outcome

Siti Hafsyah, 2021 [35]	Case report	1	37	2460	Malaysia	Female	Vaginal	N/R	Vaginal itchiness and discharge, fever, chills	Vaginal	Peritoneal fluid, pleural fluid and blood	N/R	Lethargic and grunting, cardiopulmonary arrest	Sepsis	N/R	N/R	N/R	N/R	Death
Davey 2006 [36]	Case report	1	term	3560	Canada	Female	vaginal	4 hours		Vaginal	Skin cultures	Cefazolin	Fever, erythematous scalp lesion ,irritable and fed poorly	Cellulitis	type M11 T non-typeable	Acute renal failure, respiratory distress syndrome, perforated necrotizing enterocolitis (requiring resection of necrotic bowel, a proximal jejunostomy, and distal ileal mucous fistula), recurrent infections, failure to thrive,	Ampicillin, cefuroxime, → gentamicin,meropenem and clindamycin→penicillin G	20 days	Good

																and cholestatic jaundice			
Pius, 2016 [37]	Original article	5	N/R	N/R	Nigeria	N/R	N/R	N/R		N/R	Blood	N/R	N/R	Sepsis	N/R	N/R	N/R	N/R	2 deaths
Wheeler, 1991 [38]	Case series	1	N/R	N/R	USA	Male	N/R	N/R		N/R	N/R	N/R	N/R	Pneumonia	N/R	N/R	N/R	N/R	Good
		1	N/R	N/R	USA	Male	N/R	N/R		N/R	CSF	N/R	N/R	Meningitis	N/R	N/R	N/R	N/R	Good
		1	N/R	N/R	USA	Male	N/R	N/R		N/R	N/R	N/R	N/R	Pneumonia	N/R	N/R	N/R	N/R	Good
		1	N/R	N/R	USA	Female	N/R	N/R		N/R	N/R	N/R	N/R	Pneumonia	N/R	N/R	N/R	N/R	Good
		1	N/R	N/R	USA	Male	N/R	N/R		N/R	Skin lesion	N/R	N/R	Cellulitis	N/R	N/R	N/R	N/R	N/R
Seale, 2016 [39]	observational study	94	N/R	N/R	Kenya	N/R	N/R	N/R		N/R	Blood, (2.3%) CSF, (54.7%) swab sample (wound, skin breach, or pus), (2.0%) joint aspirates, (1.5%) urine	N/R	Skin or soft tissue infection (69.9%); followed by severe pneumonia in (23.3%), of which (69%) were complicated by sepsis; then	Sepsis, meningitis	N/R	N/R	N/R	N/R	12.2% mortality

bacteremia without focus in (14.4%), (4.6%) bone and joint infections, (3.0%) meningitis, (1.6%) urinary tract infection, (0.5%) acute glomerulonephritis, (0.3%) endocarditis, (0.3%) nonspecific abdominal signs, (0.3%) necrotizing fasciitis. A total of (5.1%)

											met the criteria for strepto-coccal toxic shock syn-drome						
Givner, 1991 [31]	1	N/R	N/R	Canada	Male	N/R	N/R	Endo-metrium	Blood	N/R	N/R	Sepsis	N/R	N/R	N/R	N/R	N/R
	1	N/R	N/R	Turkey	N/R	N/R	N/R	N/R	Skin les-sion	N/R	N/R	Pyo-derma	N/R	N/R	Metroni-dazole-cepha-lothin	N/R	Good
Lehtonen, 1987 [33]	1	N/R	N/R	Turkey	N/R	N/R	N/R	N/R	Ompha-lus	N/R	N/R	Ompha-litis and conjunc-tivitis.	N/R	N/R	Metroni-dazole-cepha-lothin	N/R	Good
	17	N/R	N/R	Turkey	N/R	N/R	N/R	N/R	Ompha-lus	N/R	N/R	Ompha-litis	N/R	N/R	Metroni-dazole-cepha-lothin	N/R	Good

1. Middleton, B.; Morris, P.; Carapetis, J. Invasive group A streptococcal infection in the Northern Territory, Australia: case report and review of the literature. *Journal of paediatrics and child health* **2014**, *50*, 869-873, doi:10.1111/jpc.12659.
2. Crum, N.F.; Chun, H.M.; Gaylord, T.G.; Hale, B.R. Group A streptococcal toxic shock syndrome developing in the third trimester of pregnancy. *Infectious diseases in obstetrics and gynecology* **2002**, *10*, 209-216, doi:10.1155/s1064744902000248.
3. Mahieu, L.M.; Holm, S.E.; Goossens, H.J.; Van Acker, K.J. Congenital streptococcal toxic shock syndrome with absence of antibodies against streptococcal pyrogenic exotoxins. *The Journal of pediatrics* **1995**, *127*, 987-989, doi:10.1016/s0022-3476(95)70045-5.
4. Nieburg, P.I.; Williams, M.L. Group A beta hemolytic streptococcal sepsis in mother and infant twins. *The Journal of pediatrics* **1975**, *87*, 453, doi:10.1016/s0022-3476(75)80659-7.

5. Lequier, L.; Vaudry, W.L. Puerperal fever and neonatal pleural empyema and bacteremia caused by group A streptococcus. *The Canadian journal of infectious diseases = Journal canadien des maladies infectieuses* **1998**, *9*, 185-188, doi:10.1155/1998/470984.
6. Shinoda, Y.; Hori, T.; Sasai, H.; Ikebe, T.; Ohnishi, H. Neonatal bacteremia caused by emm type 80 group A Streptococcus: A case report. *Pediatrics international : official journal of the Japan Pediatric Society* **2020**, *62*, 1305-1306, doi:10.1111/ped.14349.
7. Verboon-Maciolek, M.A.; Krediet, T.G.; van Ertbruggen, I.; Gerards, L.J.; Fleer, A. Severe neonatal group A streptococcal disease. *European journal of pediatrics* **2000**, *159*, 450-452, doi:10.1007/s004310051305.
8. Nohara, F.; Nagaya, K.; Asai, H.; Tsuchida, E.; Okamoto, T.; Hayashi, T.; Sakata, H.; Terao, Y.; Azuma, H. Neonatal pleural empyema caused by emm type 6 group A streptococcus. *Pediatrics international : official journal of the Japan Pediatric Society* **2013**, *55*, 519-521, doi:10.1111/ped.12061.
9. Miyairi, I.; Berlingieri, D.; Protic, J.; Belko, J. Neonatal invasive group A streptococcal disease: case report and review of the literature. *The Pediatric infectious disease journal* **2004**, *23*, 161-165, doi:10.1097/01.inf.0000109887.40636.07.
10. Nutman, J.; Henig, E.; Wilunsky, E.; Reisner, S.H. Acute necrotising fasciitis due to streptococcal infection in a newborn infant. *Archives of disease in childhood* **1979**, *54*, 637-639, doi:10.1136/adc.54.8.637.
11. Thaarup, J.; Ellermann-Eriksen, S.; Stjernholm, J. Neonatal pleural empyema with group A Streptococcus. *Acta paediatrica (Oslo, Norway : 1992)* **1997**, *86*, 769-771, doi:10.1111/j.1651-2227.1997.tb08585.x.
12. Ross, L.; Tong, J. Concurrent neonatal and maternal sepsis due to invasive group A Streptococcus infection within 48 hours of birth. *Journal of paediatrics and child health* **2021**, *57*, 1698-1700, doi:10.1111/jpc.15357.
13. Sakata, H. Susceptibility and emm type of Streptococcus pyogenes isolated from children with severe infection. *Journal of infection and chemotherapy : official journal of the Japan Society of Chemotherapy* **2013**, *19*, 1042-1046, doi:10.1007/s10156-013-0617-6.
14. Sakata, H. Pediatric invasive streptococcal infection in northern and eastern regions of Hokkaido, Japan from 2010 to 2012. *Pediatrics international : official journal of the Japan Pediatric Society* **2014**, *56*, 360-363, doi:10.1111/ped.12250.
15. Timmis, A.; Parkins, K.; Kustos, I.; Riordan, F.A.; Efstratiou, A.; Carrol, E.D. Invasive group A streptococcal infections in children presenting to a paediatric intensive care unit in the North West of England. *The Journal of infection* **2010**, *60*, 183-186, doi:10.1016/j.jinf.2009.12.001.
16. Barnham, M.R.; Weightman, N.C. Bacteraemic Streptococcus pyogenes infection in the peri-partum period: now a rare disease and prior carriage by the patient may be important. *The Journal of infection* **2001**, *43*, 173-176, doi:10.1053/jinf.2001.0887.
17. Ventura, A.; Rúbio, C.; Rodrigues, D.; Silva, I.; Peres, A.; Cunha, F. Late-onset neonatal sepsis caused by group A Streptococcus - an unlikely agent. *NASCER E CRESCER - BIRTH AND GROWTH MEDICAL JOURNAL* **2019**, *28*, 26-29, doi:10.25753/BirthGrowthMJ.v28.i1.14558.
18. Saito, R.; Kerr-Liddell, R.; Paul, S.P. Late onset neonatal sepsis caused by group A streptococcus. *British journal of hospital medicine (London, England : 2005)* **2017**, *78*, 170-171, doi:10.12968/hmed.2017.78.3.170.
19. Huber, B.M. Cellulitis-adenitis in a neonate with group a streptococcal sepsis. *Klinische Padiatrie* **2014**, *226*, 82-83, doi:10.1055/s-0033-1363255.

20. Bhutta, Z.A.; Naqvi, S.H. Neonatal group A streptococcal septicemia. *The Pediatric infectious disease journal* **1990**, *9*, 528-529, doi:10.1097/00006454-199007000-00021.
21. Campbell, J.R.; Arango, C.A.; Garcia-Prats, J.A.; Baker, C.J. An outbreak of M serotype 1 group A Streptococcus in a neonatal intensive care unit. *The Journal of pediatrics* **1996**, *129*, 396-402.
22. Wilschanski, M.; Faber, J.; Abramov, A.; Isacsohn, M.; Erlichman, M. Neonatal septicemia caused by group A beta-hemolytic Streptococcus. *The Pediatric infectious disease journal* **1989**, *8*, 536-537, doi:10.1097/00006454-198908000-00015.
23. Cartwright, R.Y. Neonatal septicaemia due to group A beta-haemolytic streptococcus. *British medical journal* **1977**, *1*, 146-147, doi:10.1136/bmj.1.6054.146.
24. Barnham, M. Invasive streptococcal infections in the era before the acquired immune deficiency syndrome: a 10 years' compilation of patients with streptococcal bacteraemia in North Yorkshire. *The Journal of infection* **1989**, *18*, 231-248, doi:10.1016/s0163-4453(89)80059-3.
25. Umadevi, S.; Kali, A.; Sreenivasan, S.; Pramodhini, S.; Charles, M.V. Septic Arthritis caused by Group A Streptococcus in Newborn: An Unusual Presentation. *Journal of clinical and diagnostic research : JCDR* **2013**, *7*, 1143-1144, doi:10.7860/jcdr/2013/4852.3034.
26. Coulter, J.B.; Buchannon, C.R.; Vellodi, A.; Hart, C.A.; Sills, J.A. Group-A streptococcal infection in the newborn. *Lancet (London, England)* **1984**, *2*, 355-356, doi:10.1016/s0140-6736(84)92727-2.
27. Martic, J.; Mijac, V.; Jankovic, B.; Sekulovic, L.K.; Vasiljevic, Z.; Vuksanovic, J. Neonatal cellulitis and sepsis caused by group A streptococcus. *Pediatric dermatology* **2010**, *27*, 528-530, doi:10.1111/j.1525-1470.2010.01262.x.
28. Santos, M.S.; Ribeiro, G.S.; Oliveira, T.Q.; Santos, R.C.; Gouveia, E.; Salgado, K.; Takahashi, D.; Fontes, C.; Campos, L.C.; Reis, M.G.; et al. Burden of group A streptococcal meningitis in Salvador, Brazil: report of 11 years of population-based surveillance. *International journal of infectious diseases : IJID : official publication of the International Society for Infectious Diseases* **2009**, *13*, 456-461, doi:10.1016/j.ijid.2008.09.006.
29. Shanks, G.D.; Anderson, R.T.; Lazoritz, S.; Hemming, V.G. Bilateral neonatal group A streptococcal hydrocele infection associated with maternal puerperal sepsis. *Pediatric infectious disease* **1986**, *5*, 107-108, doi:10.1097/00006454-198601000-00022.
30. Torres-Martínez, C.; Mehta, D.; Butt, A.; Levin, M. Streptococcus associated toxic shock. *Archives of disease in childhood* **1992**, *67*, 126-130, doi:10.1136/adc.67.1.126.
31. Givner, L.B.; Abramson, J.S.; Wasilauskas, B. Apparent increase in the incidence of invasive group A beta-hemolytic streptococcal disease in children. *The Journal of pediatrics* **1991**, *118*, 341-346, doi:10.1016/s0022-3476(05)82144-4.
32. Johnson, M.P.; Rand, K.H. Group A beta-hemolytic streptococcal bacteremia and HIV infection. *Southern medical journal* **1990**, *83*, 146-149, doi:10.1097/00007611-199002000-00004.
33. Lehtonen, O.P.; Kero, P.; Ruuskanen, O.; Gaworzewska, E.T.; Hollo, O.; Erkkola, R.; Salmi, T. A nursery outbreak of group A streptococcal infection. *The Journal of infection* **1987**, *14*, 263-270, doi:10.1016/s0163-4453(87)93585-7.

34. Leonard, A.; Wright, A.; Saavedra-Campos, M.; Lamagni, T.; Cordery, R.; Nicholls, M.; Domoney, C.; Sriskandan, S.; Balasegaram, S. Severe group A streptococcal infections in mothers and their newborns in London and the South East, 2010-2016: assessment of risk and audit of public health management. *BJOG : an international journal of obstetrics and gynaecology* **2019**, *126*, 44-53, doi:10.1111/1471-0528.15415.
35. Siti Hafsyah, M.H.; Ibrahim, N.R.; Noraida, R.; Ismail, A.A.; Anani Aila, M.Z.; Hajissa, K.; Zeehaida, M. Group A Streptococcus puerperal sepsis with invasive neonatal infection: A fatal case. *The Medical journal of Malaysia* **2021**, *76*, 731-733.
36. Davey, C.; Moore, A.M. Necrotizing fasciitis of the scalp in a newborn. *Obstetrics and gynecology* **2006**, *107*, 461-463, doi:10.1097/01.aog.0000164094.02571.77.
37. Pius, S.; Bello, M.; Galadima, G.B.; Ibrahim, H.A.; Yerima, S.T.; Ambe, J.P. Neonatal septicaemia, bacterial isolates and antibiogram sensitivity in Maiduguri North-Eastern Nigeria. *The Nigerian postgraduate medical journal* **2016**, *23*, 146-151, doi:10.4103/1117-1936.190340.
38. Wheeler, M.C.; Roe, M.H.; Kaplan, E.L.; Schlievert, P.M.; Todd, J.K. Outbreak of group A streptococcus septicemia in children. Clinical, epidemiologic, and microbiological correlates. *Jama* **1991**, *266*, 533-537.
39. Seale, A.C.; Davies, M.R.; Anampiu, K.; Morpeth, S.C.; Nyongesa, S.; Mwarumba, S.; Smeesters, P.R.; Efstratiou, A.; Karugutu, R.; Mturi, N.; et al. Invasive Group A Streptococcus Infection among Children, Rural Kenya. *Emerging infectious diseases* **2016**, *22*, 224-232, doi:10.3201/eid2202.151358.
